# Construction of Freezing Injury Grade Index for Nanfeng Tangerine Plants Based on Physiological and Biochemical Parameters

**DOI:** 10.3390/plants13213109

**Published:** 2024-11-04

**Authors:** Chao Xu, Buchun Liu, Yuting Wang, Zhongdong Hu

**Affiliations:** 1 Jiangxi Key Laboratory of Horticultural Crops (Fruit, Vegetable & Tea) Breeding, Jiangxi Academy of Agricultural Sciences, Nanchang 330200, China; xuchao@jxaas.cn (C.X.); wangyuting@jxaas.cn (Y.W.); 2 Nanchang Key Laboratory of Germplasm Innovation and Utilization of Fruit and Tea, Jiangxi Academy of Agricultural Sciences, Nanchang 330200, China; 3 Institute of Environment and Sustainable Development in Agriculture, Chinese Academy of Agricultural Sciences, Beijing 100081, China; liubuchun@caas.cn

**Keywords:** freezing stress, photosynthesis, osmotic regulation, oxidative damage, low-temperature freezing stress level

## Abstract

Low-temperature freezing stress constitutes the most significant meteorological disaster during the overwintering period in the Nanfeng Tangerine (NT) production area, severely impacting the normal growth and development of the plants. Currently, the accuracy of meteorological disaster warnings and forecasts for NT orchards remains suboptimal, primarily due to the absence of quantitative meteorological indicators for low-temperature freezing stress. Therefore, this study employed NT plants as experimental subjects and conducted controlled treatment experiments under varying intensities of low-temperature freezing stress (0 °C, −2 °C, −5 °C, −7 °C, and −9 °C) and durations (1 h, 4 h, and 7 h). Subsequently, physiological and biochemical parameters were measured, including photosynthetic parameters, chlorophyll fluorescence parameters, reactive oxygen species, osmoregulatory substances, and antioxidant enzyme activities in NT plants. The results demonstrated that low-temperature freezing stress adversely affected the photosynthetic system of NT plants, disrupted the dynamic equilibrium of the antioxidant system, and compromised cellular stability. The severity of freezing damage increased with decreasing temperature and prolonged exposure. Chlorophyll (*a*/*b*) ratio (Chl (*a*/*b*)), maximum quantum yield of photosystem II (F_v_/F_m_), soluble sugar, and malondialdehyde (MDA) were identified as key indicators for assessing physiological and biochemical changes in NT plants. Utilizing these four parameters, a comprehensive score (CS) model of freezing damage was developed to quantitatively evaluate the growth status of NT plants across varying low-temperature freezing damage gradients and durations. Subsequently, the freezing damage grade index for NT plants during the overwintering period was established. Specifically, Level 1 for CS ≤ −0.50, Level 2 for −0.5 < CS ≤ 0, Level 3 for 0 < CS ≤ 0.5, and Level 4 for 0.5 < CS. The research results provide valuable data for agricultural meteorological departments to carry out disaster monitoring, early warning, and prevention and control.

## 1. Introduction

The Nanfeng Tangerine (NT, *Citrus reticulita* Blanco cv. Nanfeng Tangerine), classified within the Rutaceae family and the Citrus genus, boasts a cultivation history exceeding one thousand years and is renowned both domestically and internationally. It is distinguished by its thin skin, minimal seed content, juiciness, balanced sweet and sour flavor, unique fragrance, and rich nutritional profile [1,2]. Nanfeng County, located in Fuzhou City, Jiangxi Province, is recognized as the origin of NT. Presently, the cultivation area for NT in Nanfeng County has expanded to 107,030 acres, yielding up to 1.5 billion kilograms. Similar to other citrus species, NT plants thrive in warm and sunny climates and exhibit a marked sensitivity to low temperatures. Specifically, temperatures below 10 °C can substantially impede the growth rate of NT plants or even halt their development entirely [3]. Based on observational data from the National Ground Meteorological Station in Nanfeng County, over the past decade, the NT production area has typically experienced freezing stress within the range of −5 °C to 0 °C. However, in December 2023, the minimum temperature in this region plummeted to −8 °C, resulting in widespread freezing of orchard trees and substantial economic losses for the growers.

The photosynthetic process is highly susceptible to low-temperature stress, particularly the photo-responsive system, which can act as an indicator of the extent of plant damage induced by low temperatures [4]. Low temperature has been identified as a critical factor influencing plant photosynthesis [5,6]. Photosynthetic rate serves as a direct indicator of the functionality of the photosynthetic system, and numerous studies have demonstrated a significant decline in photosynthetic rate in response to low-temperature effects on photosynthesis [7]. The primary cause for the reduction in photosynthetic rate is attributed to not only environmental factors and stomatal conductance but also non-stomatal limiting factors. Low-temperature stress can impede chlorophyll synthesis in plants, consequently diminishing both photosynthesis and respiration [8]. Under conditions of low temperature stress, the net photosynthetic rate (P_n_), transpiration rate (T_r_), and stomatal conductance (*g*_s_) all exhibited a gradual decline, with the extent of this decline intensifying as temperatures further decreased [9]. At moderately low temperatures, the primary factor contributing to the reduction in photosynthetic rate is the limitation imposed by stomatal conductance. However, under severe low-temperature stress, the decline in photosynthetic rate is attributed to non-stomatal limiting factors [10,11]. Low temperatures significantly exacerbated damage and photoinhibition of the photosynthetic system, while markedly inhibiting non-photochemical quenching (NPQ) [12]. The NPQ response in cotton seedlings under low-temperature stress exhibits greater sensitivity compared to photochemical quenching (qP) [13]. Prolonged exposure to stress results in a decrease in the maximum photosynthetic rate of plants, with the photochemical quenching coefficient (qP) initially declining before subsequently increasing [14]. Liu et al. [15] conducted a study revealing that treatment at 5 °C and 10 °C for 8 days did not significantly impact the photosynthetic reaction system or the F_v_/F_m_. In contrast, research by Angmo et al. [16] demonstrated a decrease in F_v_/F_m_ with prolonged exposure to 5 °C, with recovery to control levels requiring 4 d. Additionally, Wu et al. [17] identified F_v_/F_o_ and F_v_/F_m_ as reliable indicators for assessing cold resistance in maize varieties.

At low temperatures, the diminished capacity of plants to utilize oxygen results in the conversion of excess oxygen into reactive oxygen species (ROS), which exert toxic effects on plant metabolism [18]. The potential toxicity of excess oxygen to aerobic organisms necessitates its immediate removal via plant antioxidant systems. In plants, there are two primary categories of ROS scavengers, namely, antioxidant enzymes and non-enzymatic antioxidants [19]. Key antioxidant enzymes in plant cells include superoxide dismutase (SOD) and catalase (CAT), among others. Under conditions of low temperature and other environmental stressors, the hydrogen peroxide scavenging system in plants is initially compromised, leading to an increase in harmful ROS [20]. Concurrently, the accumulation of hydrogen peroxide (H_2_O_2_) can inactivate SOD, thereby severely impairing the ROS scavenging system [21]. Consequently, the removal of hydrogen peroxide exerts a detoxifying effect on cellular components. Additionally, soluble sugars function as osmoregulatory substances, and plants frequently accumulate substantial quantities of these sugars in response to low temperature stress [22]. There are several mechanisms by which soluble carbohydrates enhance the cold resistance of tissues. First, they elevate cell osmotic potential, reduce cell water potential, and mitigate water loss [23]. Second, specific carbohydrates can directly interact with cellular component molecules, thereby stabilizing cell membranes and enzymes [24]. In summary, plants subjected to low-temperature stress undergo a range of physiological and biochemical adaptations to mitigate the detrimental effects of cold temperatures.

To date, research has primarily focused on the physiological and biochemical alterations in citrus plants subjected to low-temperature conditions. However, there has been a lack of development of meteorological disaster level indicators that are based on these physiological and biochemical changes to assess freezing damage. This study focuses on the characteristic citrus variety NT plants in Jiangxi Province, examining the changes in physiological and biochemical parameters under varying levels and durations of freezing damage. Characteristic parameters indicative of plant freezing damage were extracted, and a comprehensive scoring model for freezing damage was established. This model quantifies the extent of damage to NT plants under different freezing conditions and subsequently categorizes the severity of freezing damage into distinct disaster levels. The findings from this study offer valuable data to support the refinement of climate zoning and the implementation of preventive and control measures against freezing damage in the NT production region.

## 2. Results

### 2.1. The Effect of Different Degrees of Low-Temperature Freezing Process on the Gas Exchange Parameters in NT Plants

**Figure 1:**
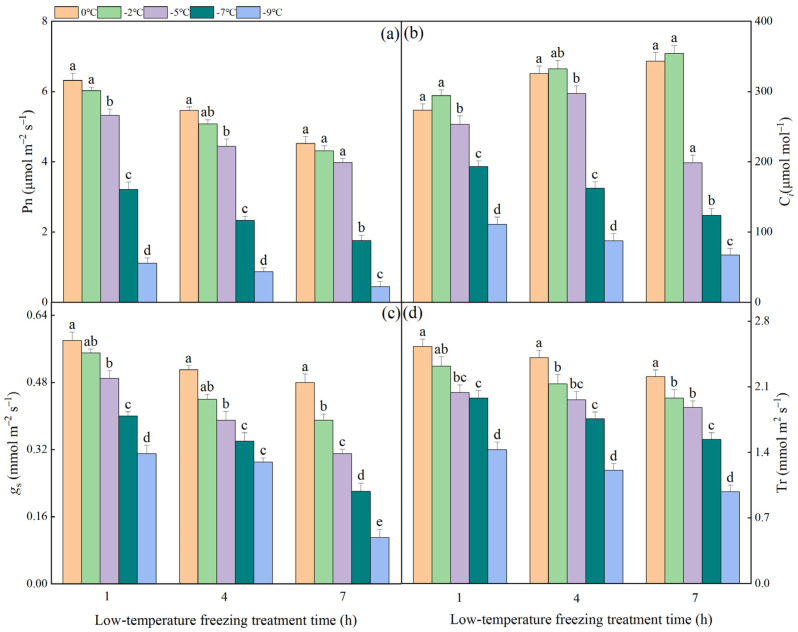
The impact of varying low-temperature freezing process on gas exchange parameters in NT plants: Panels (**a**–**d**) represent the net photosynthetic rate (P_n_), transpiration rate (T_r_), stomatal conductance (*g*_s_), and water use efficiency (WUE) of NT leaves subjected to different gradients of low-temperature freezing, respectively. Note. The data represented in the figure were the mean value derived from three replicated samples. Within the same freezing treatment duration, distinct lowercase letters signify statistically significant differences at a *p*-value less than 0.05.

The alterations in gas exchange parameters of NT plants subjected to varying gradients of low-temperature freezing processes are illustrated in Figure 1. Under freezing stress, the P_n_ of NT leaves exhibited a significant decline. This decline was more pronounced at lower temperatures and with prolonged exposure to stress. After 1 h of low-temperature freezing stress, there was no significant difference in P_n_ values between 0 °C and −2 °C. However, P_n_ values at −5 °C, −7 °C, and −9 °C were significantly lower compared to those at 0 °C. As the freezing process progressed, no significant differences in P_n_ values were observed between −2 °C, −5 °C, and 0 °C at 7 h of freezing stress. However, P_n_ values at −7 °C and −9 °C were significantly lower than those at 0 °C, exhibiting reductions of 61.06% and 90.04%, respectively. The variation pattern of C*_i_* during low-temperature freezing stress exhibited significant differences compared to that of P_n_. Specifically, at 0 °C and −2 °C, C*_i_* values demonstrated an upward trend with prolonged exposure to freezing stress. At −5 °C, C*_i_* initially increased before subsequently decreasing as freezing stress duration extended. Conversely, at −7 °C and −9 °C, C*_i_* values displayed a consistent downward trend with the extension of low-temperature freezing stress time. In contrast to C*_i_*, the variations in *g*_s_ and T_r_ during different low-temperature freezing processes exhibited a consistent pattern with P_n_.

### 2.2. The Effect of Different Degrees of Low-Temperature Freezing Process on Chlorophyll Fluorescence Parameters in NT Plants

**Figure 2:**
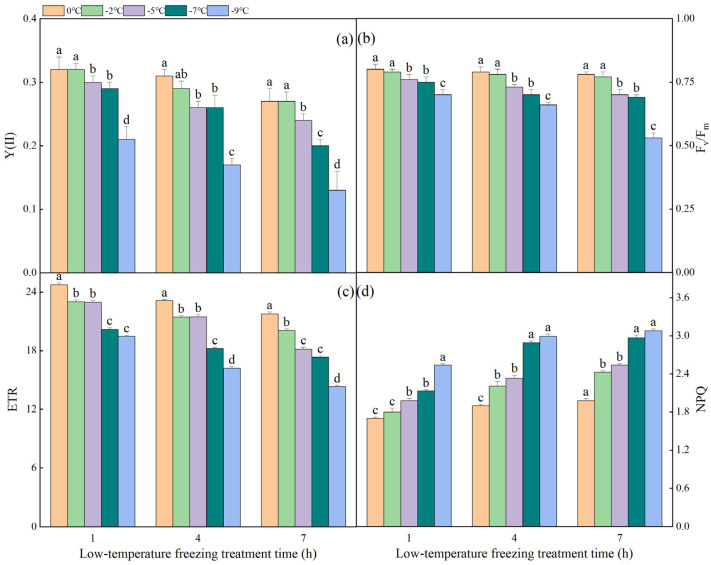
The impact of varying low-temperature freezing processes on chlorophyll fluorescence parameters in NT plants: Panels (**a**–**d**) represent the actual photosynthetic efficiency (Y(II)), maximum photosynthetic efficiency (F_v_/F_m_), electron transfer rate (ETR), and non-photochemical quenching coefficient (NPQ) in NT leaves subjected to different gradients of low-temperature freezing, respectively. Note. The data represented in the figure were the mean value derived from three replicated samples. Within the same freezing treatment duration, distinct lowercase letters signify statistically significant differences at a *p*-value less than 0.05.

Figure 2 illustrates the fluctuation pattern of chlorophyll fluorescence parameters within NT plants under varying degrees of low-temperature freezing stress. It was observed that the Y (II) values declined as the duration of stress exposure increased. During the initial hour of freezing stress, the Y (II) values between 0 °C and −2 °C did not exhibit significant differences, mirroring the pattern between −5 °C and −7 °C. However, Y (II) values at these aforementioned temperatures were significantly higher in comparison to those recorded at −9 °C. Upon exposure to 7 h of freezing stress, the Y (II) values ranging between 0 °C and −2 °C did not exhibit significant changes; however, they were notably higher than the Y (II) values at −5 °C, −7 °C, and −9 °C. The alterations in F_v_/F_m_ and ETR during varying gradients of low-temperature freezing stress were fundamentally in line with the modifications in Y (II). Specifically, they demonstrated a decrease with the reduction in temperature under identical stress duration and a decrease with the escalation of stress duration under the same stress temperature. Conversely, the alteration pattern of NPQ exhibited an inverse relationship to that of Y (II), as NPQ escalated in response to the extension of stress duration under various gradients of low-temperature freezing stress. Upon subjecting to freezing stress for a duration of 1 h, the NPQ at temperatures of −5 °C, −7 °C, and −9 °C was markedly elevated in comparison to the NPQ at 0 °C and −2 °C. Following 7 h of freezing stress, the disparity in NPQ between −2 °C and −5 °C was not significant, and similarly, the NPQ between −7 °C and −9 °C was not significantly different. However, both were superior to the NPQ at 0 °C.

### 2.3. The Effect of Different Degrees of Low-Temperature Freezing Process on the Content of Photosynthetic Pigments in NT Plants

The variations in photosynthetic pigment content of NT plants subjected to different gradients of low-temperature freezing stress are presented in Table 1. The trend in Chl *a* content indicated that, for a given duration of freezing stress treatment, a decrease in temperature corresponds to a reduction in Chl *a* content. Additionally, for a constant temperature, an increase in the duration of freezing stress treatment results in a further decline in Chl *a* content. At the 7th hour of freezing stress treatment, significant differences in Chl *a* content were observed across the various temperature gradients. Specifically, the Chl *a* content at −2, −5, −7, and −9 °C was 15.33%, 28.22%, 47.04%, and 57.14% lower than that at 0 °C, respectively. At a low temperature of −9 °C, the Chl *a* content at the 7th hour was 67.58% and 79.35% of the content measured at the 1st and 4th hours, respectively. The variation pattern of Chl *b* content under different low-temperature gradients and treatment durations was consistent with that of Chl *a* content. The range of variation in Chl (*a*/*b*) increased as the temperature decreased. Specifically, at 1 h, 4 h, and 7 h of freezing exposure, the variation ranges of Chl (*a*/*b*) under each freezing stress gradient were 2.31–2.56, 2.34–2.97, and 2.35–2.96, respectively. Additionally, at a constant freezing temperature, prolonged exposure time resulted in higher Chl (*a*/*b*) content.

### 2.4. The Effect of Different Degrees of Low-Temperature Freezing Process on Antioxidant Enzyme Activity and Osmoregulatory Substance Content in NT Plants

**Figure 3:**
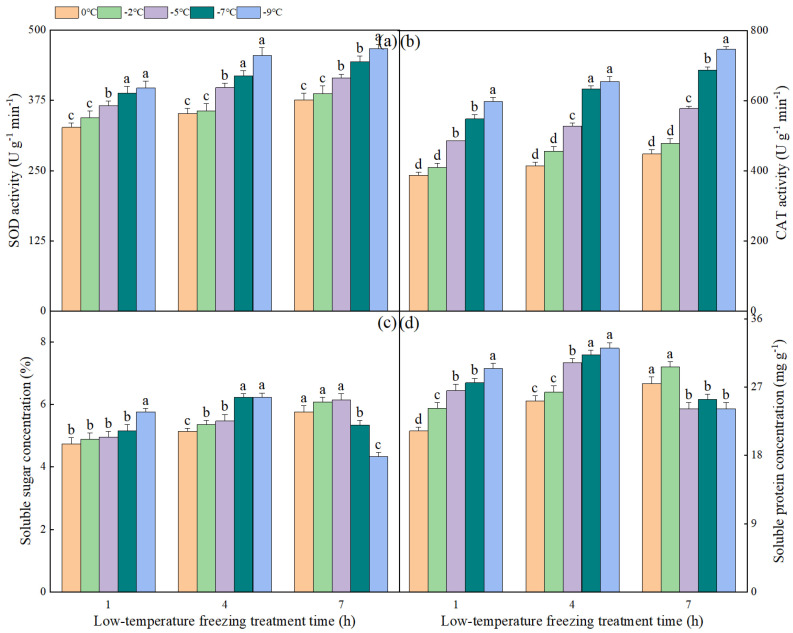
The impact of varying low-temperature freezing processes on antioxidant enzyme activity and osmoregulatory substance content in NT plants: Panels (**a**–**d**) represent the superoxide dismutase (SOD), catalase (CAT), soluble protein, and soluble sugar in NT plants subjected to different gradients of low-temperature freezing, respectively. Note. The data represented in the figure were the mean value derived from three replicated samples. Within the same freezing treatment duration, distinct lowercase letters signify statistically significant differences at a *p*-value less than 0.05.

The alterations in antioxidant enzyme activity and osmoregulatory substances in NT plants under varying gradients of low-temperature freezing conditions are illustrated in Figure 3. The activity of SOD exhibited an increase corresponding to the severity of freezing stress and the duration of exposure. After 1 h of low-temperature freezing stress, no significant difference in SOD activity was observed between −7 °C and −9 °C; however, the SOD activity at these temperatures was significantly higher compared to that at 0 °C, −2 °C, and −5 °C. After 7 h of low-temperature freezing treatment, the activity of SOD at −9 °C was significantly higher compared to its activity at 0 °C, −2 °C, −5 °C, and −7 °C, with respective increases of 24.20%, 20.67%, 12.53%, and 5.18%. The alterations in CAT activity across different gradients of low-temperature freezing stress were generally consistent with the observed changes in SOD activity. However, the trend in soluble sugar content varied across the different gradients of low-temperature freezing stress. The concentration of soluble sugars increased with the extension of exposure time at 0 °C, −2 °C, and −5 °C. After 7 h of low-temperature treatment, the soluble sugar content at 0 °C, −2 °C, and −5 °C was 21.51%, 24.28%, and 23.88% higher, respectively, compared to the levels observed after 1 h of treatment. Conversely, the soluble sugar content at −7 °C and −9 °C decreased with prolonged exposure time, reaching maximum values of 6.22% and 6.23%, respectively, after 7 h of low-temperature treatment. The variations in soluble protein and soluble sugar levels exhibited minor differences under different gradients of low-temperature freezing stress. Specifically, the soluble protein content demonstrated an upward trend with prolonged exposure at 0 °C and −2 °C. Conversely, at −5 °C, −7 °C, and −9 °C, the soluble protein content initially increased and subsequently decreased as the duration of stress increased.

### 2.5. The Effect of Different Degrees of Low-Temperature Freezing Process on the Content of Malondialdehyde and Hydrogen Peroxide in NT Leaves

**Figure 4:**
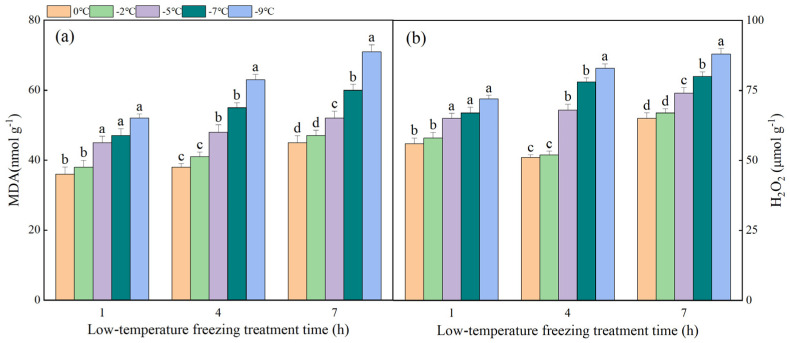
The impact of varying low-temperature freezing processes on the malondialdehyde and hydrogen peroxide content in NT plants: Panels (**a**,**b**), respectively, illustrate the malondialdehyde (MDA) and hydrogen peroxide (H_2_O_2_) content in NT plants subjected to different gradients of low-temperature freezing. Note. The data represented in the figure were the mean value derived from three replicated samples. Within the same freezing treatment duration, distinct lowercase letters signify statistically significant differences at a *p*-value less than 0.05.

The fluctuations in levels of MDA and H_2_O_2_ in NT leaves exposed to different freezing stress conditions and durations are illustrated in Figure 4. Under the same freezing stress treatment, the content of MDA increased with the prolongation of freezing stress time. Under the same duration of freezing stress treatment, the lower the temperature, the higher the content of MDA. Under the same freezing stress treatment, as the duration of stress increased, H_2_O_2_ levels initially decreased and then rose at 0 and −2 °C, while consistently increasing at −5, −7, and −9 °C. There was no statistically significant difference in H_2_O_2_ levels between −2 °C and 0 °C across varying durations of freezing stress, but H_2_O_2_ levels remained significantly elevated at −5, −7, and −9 °C compared to 0 °C. When subjected to freezing stress for 7 h, the H_2_O_2_ content reached its highest value at −5, −7, and −9 °C, which were 13.85%, 23.08%, and 35.38% higher than at 0 °C, respectively.

### 2.6. Construction of Freezing Injury Grade Index for Nanfeng Tangerine Plants Based on Physiological and Biochemical Parameters

#### 2.6.1. Extraction of Key Photosynthetic Physiological Characteristic Parameters

This research categorized the evaluated parameters into two distinct groups as follows: photosynthetic physiological parameters (P_n_, C*_i_*, *g*_s_, T_r_, Y (II), F_v_/F_m_, ETR, NPQ, Chl *a*, Chl *b*, Chl (*a*/*b*)) and biochemical parameters (SOD, CAT, soluble sugar, soluble protein, MDA, and H_2_O_2_). Table 2 illustrates the Pearson correlation analysis conducted on these physiological and biochemical parameters throughout varying gradients of low-temperature freezing processes. A clear observation from Table 2 was that Chl (*a*/*b*) exhibited a significant positive correlation with NPQ in relation to photosynthetic parameters; however, it did not demonstrate a correlation with other photosynthetic parameters. A robust positive correlation was observed among P_n_, C*_i_*, *g*_s_, T_r_, Y (II), F_v_/F_m_, ETR, Chl *a*, and Chl *b*, while a strong negative correlation was found with NPQ. Regarding physiological and biochemical parameters, a significant correlation was identified between soluble protein and soluble sugar, although no correlation was detected between these parameters and other physiological and biochemical parameters. Additionally, a significant positive correlation was noted among SOD, CAT, MDA, and H_2_O_2_. In this investigation, key parameters were chosen for evaluation of the effects of varying gradient low-temperature freezing processes on NT plants. These parameters, namely, Chl (*a*/*b*), F_v_/F_m_, soluble sugar, and MDA, were selected based on the complexity of their measurement and their representative nature. 

#### 2.6.2. Construction and Grading of Comprehensive Score for Low Temperature Freezing Injury of NT Plants

A principal component analysis was performed on the four primary physiological and biochemical parameters extracted, with the results displayed in Table 3. The cumulative variance contribution of the initial two principal components surpassed 87%, suggesting that the predominant characteristics of the four essential physiological and biochemical parameters have been accurately represented. The Principal Component scores were computed by utilizing the variance contributions and loadings furnished by PC1 and PC2 as delineated in Table 1. These scores were subsequently employed to formulate the comprehensive score (CS) of NT during the incidence of low-temperature freezing damage. The computation formula was explicitly presented in Table 4.

To facilitate application within the operations of the meteorological department, the comprehensive score has been stratified into four levels, level 1 to 4, each demarcated by a threshold of 0.5, as delineated in Table 5. An ascending value signified a progressive increase in the low-temperature freezing stress endured by NT plants.

The comprehensive score and stress level of NT plants during various gradients of low-temperature freezing injury were computed, taking into account the physiological and biochemical characteristics derived from experimental data. The corresponding results are presented in Table 6.

## 3. Discussion

Chlorophyll constitutes the fundamental material basis for energy conversion in photosynthesis, functioning as a photosensitizer essential for this process. It plays a pivotal role in plant growth and development, with its concentration directly influencing the efficacy of leaf photosynthetic activity [25,26]. Among the various stress conditions encountered during the growth and development of NT, low-temperature freezing damage is particularly prevalent. The chlorophyll content in plant leaves serves not only as an indicator of nutritional status but also as a reflective measure of the plant’s resilience to stress [27,28]. The findings of this study demonstrate that the levels of chlorophyll *a*, chlorophyll *b*, and the Chl *a*/*b* ratio in NT leaves exhibited a declining trend following exposure to a low-temperature freezing process. This decline was more pronounced with decreasing temperatures and extended treatment durations. This phenomenon may be attributed to the reduced activity of chloroplast pigment synthase, resulting in the inhibition of chlorophyll synthesis in the leaves [29]. Research [19] has demonstrated that low-temperature stress can disrupt chloroplast function and compromise chloroplast structural integrity, ultimately leading to a reduction in chlorophyll content in plant leaves [9,30]. Furthermore, under low-temperature conditions, the metabolic activity within chloroplasts is diminished, resulting in an inadequate supply of precursors necessary for chlorophyll synthesis, which further contributes to the observed decrease in chlorophyll content [29,31].

Photosynthetic organs represent the most cold-sensitive components of plants, and photosynthesis serves as a crucial determinant influencing plant growth and crop yield. The photosynthetic system within plant leaves must equilibrate the external absorption of light energy with the energy expended through plant metabolism [32]. P_n_ provides a direct measure of the photosynthetic capacity of plant leaves under specific environmental conditions. The most pronounced effect of low temperatures on photosynthesis is the reduction in P_n_ [33]. Soualiou et al. [34] demonstrated that both stomatal and non-stomatal factors contribute to the reduction in photosynthetic rate induced by low temperatures. When P_n_, *g*_s_, and T_r_ decrease simultaneously, the *C_i_* also decreases, suggesting a predominance of stomatal factors; conversely, if P_n_, *g*_s_, and T_r_ decrease while *C_i_* increases, this indicates that non-stomatal factors are impeding CO_2_ utilization, leading to an accumulation of *C_i_* [9,35,36]. In the present study, P_n_, *g*_s_, and T_r_ continued to decline under treatments at 0 °C and −2 °C, while *C_i_* continued to increase. This suggests that the reduction in P_n_ under treatment conditions of 0 °C and −2 °C is attributable to stomatal factors. Under the −5 °C treatment, P_n_, *g*_s_, and T_r_ exhibited a continuous decline, whereas the *C_i_* initially increased and subsequently decreased, attaining its peak value after 4 d of exposure to low temperatures. This suggests that under the −5 °C treatment conditions, the initial reduction in P_n_ within the first four hours was attributable to stomatal factors. However, beyond this period, the decline in P_n_ was not due to decreased stomatal conductance limiting CO_2_ supply but rather to non-stomatal factors such as limitations in photochemical activity, ribulose—1,5-bisphosphate (RuBP) carboxylation, and inorganic phosphorus availability, which impeded CO_2_ utilization and led to CO_2_ accumulation in the intercellular spaces. Under the treatment conditions of −7 °C and −9 °C, P_n_, *g*_s_, and T_r_ decreased, accompanied by a reduction in *C_i_*, indicating that the decrease in P_n_ was influenced by both stomatal factors.

In comparison to apparent photosynthetic indicators, chlorophyll fluorescence parameters provide a more accurate reflection of the intrinsic characteristics of plant photosynthetic systems [37]. The PSII reaction center constitutes a critical component of the photosynthetic apparatus, playing a pivotal role in the conversion of light energy during photosynthesis [38]. The parameter F_v_/F_m_, representing the maximum photochemical quantum yield of PSII, is widely employed to assess the primary light energy conversion efficiency of PSII in plant leaves. It serves as a crucial indicator for assessing plant photosynthetic performance and is presently the most extensively utilized photosynthetic parameter [39,40]. Various gradient low-temperature treatments led to a significant reduction in the F_v_/F_m_ of NT leaves, while Y(II) and ETR also exhibited substantial declines with extended treatment duration, corroborating previous research findings in grapevine [41]. Given that low temperatures exert a significantly greater influence on carbon assimilation processes compared to other photosynthetic mechanisms, the observed reduction in F_v_/F_m_ can be attributed to the diminished activity of Rubisco and its activators under cold stress [42]. This reduction in Rubisco activity impairs the leaves’ capacity to assimilate CO_2_, thereby decreasing the demand for ATP and NADPH in the Calvin cycle. Consequently, this leads to feedback regulation of the oxidation–reduction state of PSII, culminating in the accumulation of excess light energy [43]. Y (II) represents the actual photosynthetic quantum yield of Photosystem II (PSII), and ETR indicates the apparent efficiency of electron transfer under prevailing light intensity conditions [17]. A reduction in Y (II) and ETR signifies an impediment in the electron transfer process from QA to QB [44]. NPQ quantifies the proportion of light energy absorbed by the PSII reaction center antenna pigments that is not utilized for photosynthetic electron transfer. Instead, this excess light energy is dissipated as heat, thereby reflecting the plant’s capacity for thermal dissipation of surplus light energy and its photoprotective capability [45]. The marked reduction in Y (II) and ETR, coupled with the elevation in NPQ, suggests that low temperatures impair the re-oxidation capacity of the primary electron acceptor QA. This impairment results in diminished electron transfer activity within PSII and a subsequent decline in carbon assimilation [25]. Consequently, the transfer of electrons carried by the reduced state QA to subsequent electron transporters becomes increasingly challenging, thereby obstructing electron transfer. As a result, a greater proportion of absorbed light energy is dissipated through non-photochemical pathways, ultimately inflicting damage on the photosynthetic system and leading to a decrease in photosynthetic efficiency, which in turn affects normal plant growth [25].

Following exposure to low-temperature stress, plants exhibit a continuous accumulation of reactive oxygen species (ROS), which in turn induces membrane lipid peroxidation [46]. MDA is a principal by-product of this peroxidation process, contributing to enhanced membrane permeability, intracellular electrolyte leakage, and elevated relative conductivity [47]. The findings of this study demonstrated that MDA levels in NT leaves escalated as the intensity of low temperature decreases and the duration of stress increases, suggesting that low-temperature freezing damage compromises the stability of NT leaf cell membranes, resulting in pronounced membrane lipid peroxidation, and this damaging effect intensifies with the degree and duration of freezing damage. SOD and CAT are critical antioxidant enzymes in plant cells that decompose toxic free radicals and hydrogen peroxide, thereby maintaining cellular ROS levels and playing a significant role in preventing cellular senescence [44]. The upregulation of these protective enzymes constitutes a self-defense mechanism in plants under adverse environmental conditions. In this study, during exposure to low-temperature freezing, the activities of SOD and CAT in NT leaves exhibited a continuous increase. This enhancement facilitated the accelerated removal of harmful hydrogen peroxide from plant cells, thereby mitigating the damage caused by free radicals [48].

Osmotic regulation constitutes a critical physiological mechanism enabling plants to withstand adverse conditions [49]. By synthesizing osmoregulatory substances, such as soluble sugars and proteins, plants actively manage cellular osmotic balance, thereby enhancing their resilience against both biotic and abiotic stressors [50]. Soluble sugars, in particular, contribute to increased cellular fluid concentration, reduced cellular water potential, and enhanced water retention capacity, which collectively lower the freezing point. Additionally, these sugars can induce protein synthesis, thereby improving the cold resistance of fruit trees [51]. Guan et al. [52] demonstrated that the cold resistance of pomegranate is correlated with its soluble sugar content. In the present study, it was observed that during exposure to temperatures of 0 °C, −2 °C, and −5 °C, the soluble sugar content gradually increased with decreasing temperature. However, at temperatures of −7 °C and −9 °C, the soluble sugar content initially increased during the first four hours, followed by a subsequent decrease. This decline may be attributed to the disruption of the osmotic regulation metabolic system, which impedes the synthesis of related enzymes [52]. Soluble proteins display analogous patterns. In summary, the accumulation of osmoregulatory substances in response to environmental stress constitutes a defensive mechanism employed by plants to counteract adverse conditions. However, when the tolerance threshold is surpassed, plants rapidly lose their self-protective capacity, leading to damage and potentially irreversible harm [53].

## 4. Materials and Methods

### 4.1. Experimental Materials and Treatments

This study was conducted at the Jiangxi Academy of Agricultural Sciences using three-year-old potted seedlings of the NT variety, a prominent citrus species in Jiangxi Province. The NT seedlings were planted in containers measuring 25 cm (height) × 50 cm (upper diameter) × 50 cm (lower diameter), filled with soil sourced from the NT Orchard with a pH value of approximately 6.2. The soil contained 84 mg kg^−1^ of available nitrogen, 37 mg kg^−1^ of available phosphorus, and 123 mg kg^−1^ of available potassium. The soil moisture content was maintained at 60–65% during the experiment.

Healthy and consistent NT seedlings were selected for controlled experiments. Next, they were then subjected to simulated nighttime frost meteorological scenes in the climate chamber (LRX-2000E-LED, Prandtl, China). The temperature treatments included −2 °C, −5 °C, −7 °C, and −9 °C, with durations of 1 h, 4 h, and 7 h. The temperature of 0 °C was taken as the control. During the whole experiment, the artificial climate chamber maintained a photosynthetic effective radiation (PAR) of 0 μmol m^−2^ s^−1^ and a relative humidity of 50% to 60%. Each treatment was replicated three times, with five pots of seedlings in each group.

### 4.2. Determination of Physiological and Biochemical Parameters

#### 4.2.1. Measurement of Gas Exchange Parameters

The LI-6400 portable photosynthesis instrument (LI-COR, Lincoln, NE, USA) was utilized for the assessment of leaf photosynthetic parameters. Three seedlings exhibiting uniform growth were chosen for each treatment, with three middle mature leaves selected from each seedling for measurement. The light intensity of the red and blue light source within the leaf chamber was adjusted to 1000 μmol m^−2^ s^−1^ to ensure light saturation of the NT leaves, while the CO_2_ concentration was set at 400 μmol mol^−1^. Photosynthetic parameters, including leaf net photosynthetic rate (P_n_, μmol m^2^ s^−1^), transpiration rate (T_r_, mmol m^2^ s^−1^), intercellular carbon dioxide concentration (*C_i_*, μmol mol^−1^), and stomatal conductance (*g*_s_, mmol m^2^ s^−1^), were assessed between the hours of 9:00 and 11:00 a.m. [9].

#### 4.2.2. Determination of Chlorophyll Fluorescence Parameters

The leaves utilized for measuring gas exchange parameters were also selected for the measurement of chlorophyll fluorescence parameters in NT seedlings using the pulse-amplitude modulation (PAM) fluorometer (PAM 2500; Heinz Walz GmbH, Effeltrich, Germany). A specialized dark-adapted leaf clip, equipped with a fluorometer, was employed for a 30-min dark adaptation period. The actual photosynthetic efficiency [Y(II)], maximum photosynthetic efficiency (Fv/Fm), electron transfer rate (ETR), non-photochemical quenching coefficient (NPQ) of PSII, and other chlorophyll fluorescence parameters were automatically recorded by the instrument [54].

#### 4.2.3. Determination of Photosynthetic Pigment Content

The concentration of photosynthetic pigments in leaves was assessed through the utilization of an 80% acetone extraction technique. The same leaves employed for the determination of gas exchange parameters were selected, weighed at 0.100 g, and subjected to the addition of 10 mL of extraction solution, followed by a 48 h extraction period. The resulting extracts exhibited colorimetric properties at wavelengths of 663 nm, 645 nm, and 440 nm, enabling the calculation of chlorophyll *a* (Chl *a*) and chlorophyll *b* (Chl *b*) [3].

#### 4.2.4. Determination of Antioxidant Enzyme Activity and Osmoregulatory Substances

For this, 0.5 g of leaves (the leaves taken are the same as those measured by chlorophyll fluorescence) were measured and transferred to a mortar. Then, 5 mL of phosphate buffer with a pH of 7.8 was added and a small quantity of quartz sand for grinding assistance. The mixture was ground in an ice bath, and then the homogenate was transferred to a centrifuge tube and centrifuged at a high speed for a duration of 20 min. Subsequently, the supernatant (referred to as the enzyme solution) was decanted into a test tube and stored at a temperature range of 0–4 °C for future use. The activity of superoxide dismutase (SOD) was assessed using the nitrogen blue tetrazole (NBT) colorimetric method [55], while catalase (CAT) activity was determined using the UV absorption method [56].

The quantification of soluble protein content was conducted using the Coomassie Brilliant Blue G-250 assay, as described by Doganlar et al. [57]. Soluble sugars were extracted thrice using 80% ethanol in an 80 °C water bath, and their contents were subsequently determined following the protocol established by Leach and Braun [58].

#### 4.2.5. Determination of Malondialdehyde and Hydrogen Peroxide

The concentration of malondialdehyde (MDA) was quantified using the thiobarbituric acid method. Specifically, 0.75 mL of supernatant enzyme extract was combined with 1 mL of thiobarbituric acid for analysis. The resulting mixture was subjected to heat treatment in a boiling water bath for 30 min, followed by cooling to room temperature and subsequent centrifugation at 10,000 r min^−1^ for 15 min to obtain the supernatant. Absorbance readings at wavelengths of 450, 532, and 600 were taken, and the MDA content in the leaves was subsequently calculated [59]. The hydrogen peroxide content (H_2_O_2_) was quantified using the method outlined by Xu et al. [60]. A 0.5 g leaf sample was combined with 5 mL of 0.1% trichloroacetic acid solution, ground in liquid nitrogen, and centrifuged at 10,000 r min^−1^ for 10 min. Subsequently, 0.5 mL of the supernatant was added to 5 mL of 10 mmol L^−1^ sodium phosphate buffer and 1 mL of potassium iodide solution, and the absorbance was measured at 180 nm. The reaction was allowed to proceed in the dark at 28 °C for 1 h before calculating the H_2_O_2_ content.

### 4.3. Data Analysis and Processing

The statistical analysis and data visualization presented in this article were conducted using Microsoft Excel 2023 and Origin software systems (OriginLab, Northampton, MA, USA). To assess statistical significance, Duncan’s multiple comparison test was employed, with a significance threshold set at 0.05. The measured parameters are expressed as mean ± standard deviation.

The principal component analysis (PCA) method is employed to reduce the dimensionality of high-dimensional variable spaces, with the objective of minimizing information loss. This technique transforms multiple indicators into a smaller set of comprehensive indicators, known as principal components. Each principal component encapsulates the majority of the information from the original variables, ensuring that there is no redundancy in the data representation [61].

The procedure for PCA can be delineated as follows: Let matrix X consist of n samples (x_1_, x_2_, …, x_n_), where each sample is characterized by m-dimensional variables. To mitigate discrepancies arising from variations in dimensions or magnitudes, X is subjected to standardization. The resultant standardized matrix, denoted as X*, is presented in Equation (1).

(1) $$X^{\prime }=\left[\begin{array}{cccc} {x^{\prime }}_{11} & {x^{\prime }}_{12} & \cdots & {x^{\prime }}_{1n}\\ {x^{\prime }}_{21} & {x^{\prime }}_{22} & \ldots & {x^{\prime }}_{2n}\\ \vdots & \vdots & \ddots & \vdots \\ {x^{\prime }}_{m1} & {x^{\prime }}_{m2} & \cdots & {x^{\prime }}_{mn} \end{array}\right]$$

Computing the correlation coefficient matrix for Equation (1). Subsequently, determine the eigenvalues (λ_n_). The λ_n_ encapsulates the original information content represented by the nth principal component. Furthermore, the variance contribution rate (w*_i_*) is illustrated in Equation (2).

(2) $$w_{n}=\frac{\lambda_{n}}{\sum_{n=1}^{k}\lambda_{n}}$$

Arrange the weights (w*_i_*) obtained in the preceding step in descending order. Select the corresponding principal components when the cumulative variance contribution rate reaches or exceeds 85%. Subsequently, calculate the loadings (b_mn_) as delineated in Equation (3). A higher absolute value of b_mn_ signifies a more substantial influence on the evaluation outcomes.

(3) $$b_{\mathrm{mn}}=X_{\mathrm{mn}}\sqrt{\lambda_{k}}$$

## 5. Conclusions

This study investigated the dynamic alterations in 18 photosynthetic physiological and biochemical parameters in Nanfeng Tangerine (NT) plants subjected to five low-temperature conditions: 0 °C, −2 °C, −5 °C, −7 °C, and −9 °C. The parameters chlorophyll *a*/*b* ratio (Chl *a*/*b*), maximum quantum yield of PSII (F_v_/F_m_), soluble sugar content, and malondialdehyde (MDA) were identified as key indicators of low-temperature freezing damage in NT plants. A comprehensive score (CS) was developed based on the above four photosynthetic fluorescence characteristics, providing a quantitative assessment of the severity of stress on the photosynthetic system and the plants’ adaptive responses to low-temperature conditions. Finally, the freezing damage of NT plants was categorized into four distinct levels based on the CS value, specifically levels 1 to 4, with each level demarcated by a threshold increment of 0.5. The research results provide theoretical support for the assessment and prevention of low-temperature freezing damage to NT plants.

## Figures and Tables

**Table 1:** The effect of different degrees of low-temperature freezing process on the content of photosynthetic pigments in NT plants.

Treatment Time (h)	Freezing Temperature (°C)	Chl *a* (mg g^−2^)	Chl *b* (mg g^−2^)	Chl (*a*/*b*)
1	0	3.08 ± 0.01 a	1.33 ± 0.01 a	2.31 ± 0.01 c
	−2	3.00 ± 0.02 a	1.24 ± 0.02 b	2.42 ± 0.01 c
	−5	2.75 ± 0.02 b	1.11 ± 0.02 c	2.48 ± 0.02 c
	−7	2.13 ± 0.01 c	0.85 ± 0.01 c	2.51 ± 0.01 b
	−9	1.82 ± 0.01 d	0.71 ± 0.01 c	2.56 ± 0.01 a
4	0	3.02 ± 0.01 a	1.27 ± 0.01 a	2.34 ± 0.02 c
	−2	2.99 ± 0.01 a	1.11 ± 0.02 a	2.69 ± 0.01 b
	−5	2.53 ± 0.02 b	0.86 ± 0.01 b	2.94 ± 0.02 a
	−7	1.99 ± 0.01 c	0.67 ± 0.01 c	2.97 ± 0.01 a
	−9	1.55 ± 0.01 d	0.51 ± 0.01 c	3.04 ± 0.02 a
7	0	2.87 ± 0.01 a	1.22 ± 0.02 a	2.35 ± 0.02 c
	−2	2.43 ± 0.01 b	1.01 ± 0.01 b	2.41 ± 0.01 b
	−5	2.06 ± 0.02 c	0.85 ± 0.01 b	2.42 ± 0.01 b
	−7	1.52 ± 0.01 d	0.61 ± 0.01 b	2.49 ± 0.02 b
	−9	1.23 ± 0.01 e	0.33 ± 0.01 c	3.24 ± 0.01 a

Note. The data represented in the table were the mean value derived from three replicated samples. Within the same freezing treatment duration, distinct lowercase letters signify statistically significant differences at a *p*-value less than 0.05.

**Table 2:** Pearson correlation analysis of physiological and biochemical parameters in NT plants under different gradients of the low-temperature freezing process.

.	P_n_	C*_i_*	*g*_s_	T_r_	Y (II)	F_v_/F_m_	ETR	NPQ	Chl *a*	Chl *b*	Chl (*a*/*b*)	SOD	CAT	Soluble Sugar	Soluble Protein	MDA	H_2_O_2_
P_n_	1	0.881 **	0.908 **	0.955 **	0.918 **	0.857 **	0.913 **	−0.903 **	0.956 **	0.949 **	−0.409	−0.907 **	−0.938 **	−0.198	−0.446	−0.924 **	−0.885 **
C*_i_*		1	0.790 **	0.863 **	0.807 **	0.835 **	0.797 **	−0.774 **	0.905 **	0.877 **	−0.319	−0.794 **	−0.891 **	0.016	−0.159	−0.838 **	−0.836 **
*g*_s_			1	0.934 **	0.937 **	0.924 **	0.962 **	−0.941 **	0.950 **	0.953 **	−0.322	−0.949 **	−0.962 **	−0.129	−0.255	−0.955 **	−0.890 **
T_r_				1	0.961 **	0.927 **	0.930 **	−0.912 **	0.947 **	0.963 **	−0.421	−0.927 **	−0.943 **	−0.115	−0.388	−0.961 **	−0.907 **
Y (II)					1	0.932 **	0.925 **	−0.895 **	0.916 **	0.916 **	−0.318	−0.931 **	−0.911 **	−0.106	−0.268	−0.958 **	−0.890 **
F_v_/F_m_						1	0.908 **	−0.853 **	0.895 **	0.923 **	−0.316	−0.899 **	−0.922 **	0.073	−0.139	−0.950 **	−0.886 **
ETR							1	−0.955 **	0.947 **	0.949 **	−0.384	−0.967 **	−0.948 **	−0.254	−0.323	−0.964 **	−0.913 **
NPQ								1	−0.925 **	−0.947 **	0.524 *	0.955 **	0.948 **	0.343	0.435	0.942 **	0.900 **
Chl *a*									1	0.973 **	−0.344	−0.950 **	−0.976 **	−0.168	−0.317	−0.956 **	−0.944 **
Chl *b*										1	−0.511	−0.957 **	−0.984 **	−0.161	−0.400	−0.964 **	−0.930 **
Chl (*a*/*b*)											1	0.455	0.433	0.451	0.742 **	0.400	0.396
SOD												1	0.963 **	0.274	0.392	0.981 **	0.954 **
CAT													1	0.140	0.315	0.972 **	0.942 **
Soluble Sugar														1	0.681 **	0.145	0.236
Soluble Protein															1	0.320	0.357
MDA																1	0.962 **
H_2_O_2_																	1

Note. A double asterisk (**) denoted a significant correlation at the 0.01 level, while a single asterisk (*) signified a significant correlation at the 0.05 level. P_n_, C*_i_*, *g*_s_, T_r_, Y (II), F_v_/F_m_, ETR, NPQ, Chl *a*, Chl *b*, SOD, CAT, MDA, and H_2_O_2_ represented net photosynthetic rate, intercellular carbon dioxide concentration, stomatal conductance, transpiration rate, actual photosynthetic efficiency, maximum photosynthetic efficiency, electron transfer rate, non-photochemical quenching coefficient, chlorophyll *a*, chlorophyll *b*, superoxide dismutase, catalase, malondialdehyde, and hydrogen peroxide, respectively.

**Table 3:** Variance contribution and loadings of key parameters in principal components analysis.

Principal Component Number		PC1	PC2
Eigenvalue		2.216	1.282
Percentage of Variance (%)		55.406	32.052
Cumulative Percentage of Variance (%)		55.406	87.458
Loadings	F_v_/F_m_	−0.599	0.382
	Chl (*a*/*b*)	0.440	0.481
	Soluble sugar	0.203	0.759
	MDA	0.637	−0.215

Note. F_v_/F_m_ and MDA were representative of the maximum photosynthetic efficiency and malondialdehyde, respectively. PC1 and PC2 were indicative of the two principal components that contribute the most significantly to variance.

**Table 4:** Calculation formula for principal component scores of key parameters and comprehensive score in NT plants under different gradients of low-temperature freezing process.

PCA Scores	Formulas
PC1 Score	SPC_1_ = −0.599 × F_v_/F_m_ + 0.440 × Chl (*a*/*b*) + 0.203 × soluble sugar + 0.637 × MDA
PC2 Score	SPC_2_ = 0.382 × F_v_/F_m_ + 0.481 × Chl (*a*/*b*) + 0.759 × soluble sugar −0.215 × MDA
Comprehensive score	CS = 0.554 × SPC_1_ + 0.321 × SPC2

**Table 5:** Low temperature freezing stress level of NT plants.

Comprehensive Scores for Freezing Injury	Low Temperature Freezing Stress Level
CS ≤ −0.50	1
−0.5 < CS ≤ 0	2
0 < CS ≤ 0.5	3
0.5 < CS	4

Note: CS denoted the comprehensive score for NT plants’ freezing injury. An elevated stress level signified a heightened severity of freezing stress.

**Table 6:** The comprehensive score and stress level of NT plants in this research.

Treatment Time (h)	1		4		7	
Freezing Temperature (°C)	Score	Level	Score	Level	Score	Level
0	−1.01	1	−0.72	1	−0.25	2
−2	−0.74	1	−0.07	2	0.03	3
−5	−0.45	2	0.28	3	0.52	4
−7	−0.26	2	0.38	3	1.10	4
−9	0.06	4	1.13	4	1.32	4

Note: Score denoted the comprehensive score for NT plants’ freezing injury. Level: Stress level of NT plants.

## Data Availability

Data are available upon request.
